# Effect of dietary fenugreek seeds on the antioxidant status of gilthead seabream (*Sparus aurata* L.)

**DOI:** 10.1007/s13197-024-06096-9

**Published:** 2024-10-17

**Authors:** Antonia M. Jiménez-Monreal, Francisco A. Guardiola, M. Antonia Murcia, Diana Ceballos-Francisco, M. Ángeles Esteban, Magdalena Martínez-Tomé

**Affiliations:** 1 Department of Food Science, Faculty of Veterinary, Regional Campus of International Excellence “Campus Mare Nostrum”, University of Murcia, Campus de Espinardo, Murcia, 30100 Spain; 2 CIBER: CB12/03/30038 Fisiopatologia de la Obesidad y la Nutricion, CIBEROBN, Instituto de Salud Carlos III (ISCIII), Murcia, Spain; 3 Department of Cell Biology and Histology, Faculty of Biology, Regional Campus of International Excellence “Campus Mare Nostrum”, University of Murcia, Murcia, 30100 Spain

**Keywords:** Dietary fenugreek, Growth, Status antioxidant, Gilthead seabream (*Sparus aurata* L.), Aquaculture

## Abstract

The goal of this work was to determine the effects of dietary supplementation with fenugreek seeds on the oxidative status of gilthead seabream. Fish were fed four different diets for 8 weeks: three groups of fish were fed diets containing 1%, 5% or 10% (w/w) fenugreek seeds, while the control group was fed a diet without fenugreek. At the end of the experiment, the expression gene of the main antioxidant enzymes (superoxide dismutase, catalase and glutathione reductase) was measured in the fish liver. Supplementation with fenugreek seeds at 10% significantly increased the growth of fish while different effects on gene expression were detected. An increase in H_2_O_2_ and OH⋅ scavenging ability and oxidative stability was observed in fish fed fenugreek-supplemented diets. In the present study, an 8-week supplementation of the diet of gilthead seabream specimens with fenugreek seeds resulted in an improvement of fish growth and antioxidant status (ROS scavenging, catalase and oil oxidative stability). These results afford a new perspective on the use of spices with medicinal properties as a supplement in fish feed in order to improve their growth and antioxidant status.

## Introduction

Gilthead seabream (*Sparus aurata*) is the only species of seabream which is currently cultivated on a large scale. It is common throughout the Mediterranean Sea and is also found along the Eastern Atlantic coasts, from the United Kingdom to the Canary Islands. The EU is by far the biggest producer worldwide (followed by Turkey) and, within the EU, Greece is the largest producer, followed by Spain. The demand for fresh fish has increased significantly in recent decades (Simat et al. 2012). Primarily sold fresh, its shelf-life is estimated at around two weeks (Cakli et al. 2007).

The regular consumption of fish is known to provide many benefits for human health because of its high content of essential PUFA of the n-3 family and its content of high-quality proteins, vitamins and minerals (Mnari et al. 2012); however, the high PUFA content makes fish very susceptible to lipid oxidation. Lipid hydroperoxides, the primary products of lipid oxidation, damage the cellular biomembranes, and, to protect the cells from oxidative damage, fish have developed their own endogenous antioxidant defence system, which can be increased by including exogenous antioxidant compounds in the diet. For example, supplementing the diet with natural antioxidants such as thyme and rosemary has proved effective in several species (Alvarez et al. 2012; Gao et al. 2013; Mohseni and Ozorio 2014).

*Trigonella foenum-graecum* (fenugreek) is a plant of the Leguminaceae family, whose seeds have long been used by Asian, African and Mediterranean populations in their daily diet. Fenugreek seed is a rich source of calcium, iron, carotenes and other phytochemicals (Benayad et al. 2014) and, for this reason, it is used as a dietary antioxidant supplement for its antidiabetic, hypocholesterolaemic, antimicrobial, anti-inflammatory and anti-cancer properties (Benayad et al. 2014). In a previous work, our team evaluated the effects of fenugreek on gilthead seabream immune status and growth performance after 4 weeks of treatment (Awad et al. 2015). The results confirmed that fenugreek promotes the growth and stimulates the tested immune parameters. In order to investigate other potential beneficial effects of incorporating fenugreek seeds in the fish diet, we evaluated the effect of fenugreek seeds-supplemented diets on the antioxidant status of farmed gilthead seabream.

## Materials and methods

### Materials

All the chemicals used were of chromatographic grade and were purchased from Sigma Chemical Co. (Poole, Dorset UK). Propyl gallate (E-310), a food additive widely used in the food industry and compiled in the Alimentarius Codex (WHO 1999), was used as antioxidant standard (at the permitted concentration of 100 mg g^− 1^).

### Diets preparation

Fenugreek seeds (*T. foenum-graecum*) were collected from a market in Cairo (Egypt) and crushed. A commercial pellet diet (Skretting, Spain) was also crushed before being mixed with 10 g (1%), 50 g (5%) or 100 g (10%) kg^− 1^ (w/w) of fenugreek seed powder and tap water. The diets were re-made into pellets, allowed to dry and stored at 4 °C until use. The ingredients of the control diet were fish flour, fish oil, vegetable oils, wheat gluten, cereal grains, legumes grains, oilseeds, vitamins and minerals. The proximate composition was: crude protein 45.5%, crude fat 19.0%, ash 6.2%, cellulose 4.0%, P total 0.95% and Energy 17.67 MJ kg^− 1^. The control diet (0%) was prepared in the same way but adding only water.

### Fish

Eighty (45.47 ± 9.5 g weight and 13.96 ± 0.9 cm length) specimens of the marine teleost gilthead seabream (*S. aurata*, L.) were obtained from a local farm (Murcia, Spain), and acclimatized for 2 weeks in re-circulating seawater aquaria (250 L) at the Marine Fish Facility of the University of Murcia. The water temperature was maintained at 20 ± 2 °C with a flow rate of 900 L h^− 1^ and 28% salinity. The photoperiod was adjusted to 12 h light: 12 h dark. Fish were fed twice daily with a commercial pellet diet (Skretting, Spain) at a rate of 2% body weight day^− 1^ (Guardiola et al. 2016). All experimental protocols were approved by the Ethical Committee of the University of Murcia and were carried out in accordance with EU Directive 2010/63/EU for animal experiments.

### Experimental design and sampling

Eighty fish were randomly distributed into eight identical tanks (10 fish per tank, 2 replicates, 20 fish per group) where the following groups were established: (1) control, non-supplemented diet (0%); (2) 1% diet supplemented with fenugreek seeds; (3) 5% diet supplemented with fenugreek seeds; and (4) 10% diet supplemented with fenugreek seeds. The fish were fed at a rate of 2% body weight day^− 1^ for 8 weeks. The selected specimens were sacrificed by using an overdose of MS-222 (Sandoz, 100 mg L^− 1^ water). The fish were weighed and measured, and then blood samples were collected from the caudal vein with an insulin syringe and the liver and muscle were dissected. The blood samples were left to clot at 4 °C for 4 h and later the serum was collected after centrifugation (10,000 *× g*, 5 min, 4 °C) and stored at -80 °C until use. Fragments of liver were stored in TRIzol Reagent (Invitrogen) at -80 ºC for gene expression analysis (Guardiola et al. 2016).

### Growth performance

The body weight and length of each fish was measured before the trial, and growth was calculated by obtaining the weight gain (WG%), specific growth rate (SGR), and condition factor (CF), according to Guzmán-Villanueva et al. (2014):

- WG% = ((Wf ─ Wi) Wi-1) × 100.
- SGR = [(Ln final weight ─ Ln initial weight) number of days^-1^] ×100.
- CF = (weight length^-3^) × 100.

Wi: initial weight; Wf: final weight.

### Antioxidant status

The anti-oxidant status of serum from specimens fed the different experimental diets was measured spectrophotometrically (BOECO, S-22 UV/VIS) by means of a BAP (biological antioxidant potential) test according to the procedure outlined by the manufacturer (Diacron International S.R.L, Grosseto, Italy). Briefly, 50 µL of R2 reagent (ferric salt) was transferred to 1 mL of R1 reagent (a particular thiocyanate derivative), mixed gently and read on a photometer at a wavelength of 505 nm. Then, 10 µL of reagent blank, calibrator or serum sample were added to the cuvette, mixed gently and read on the above-mentioned photometer. The values were expressed as µmol antioxidant substance L^− 1^ of vitamin C, which was used as an iron-reducing agent reference.

### Gene expression analysis

After 8 weeks of feeding, total RNA was extracted from 0.5 g of seabream liver samples using TRIzol Reagent (Chomczynski 1993). It was then quantified, and the purity was assessed by spectrophotometry; the 260:280 ratios were 1.8-2.0. The RNA was then treated with DNase I (Promega) to remove genomic DNA contamination. Complementary DNA (cDNA) was synthesized from 1 µg of total RNA using the SuperScript III reverse transcriptase (Invitrogen) with an oligo-dT18 primer. The expression of the selected genes was analysed by real-time PCR, which was performed with an ABI PRISM 7500 instrument (Applied Biosystems) using SYBR Green PCR Core Reagents (Applied Biosystems). Reaction mixtures (containing 10 µL of 2 x SYBR Green supermix, 5 µL of primers (0.6 µM each) and 5 µL of cDNA template) were incubated for 10 min at 95 ºC, followed by 40 cycles of 15 s at 95 ºC, 1 min at 60 ºC, and finally 15 s at 95 ºC, 1 min at 60 ºC and 15 s at 95 ºC. For each mRNA, gene expression was corrected by the geometric mean of the Ct value of the elongation factor 1α (*ef1a*), *β*-actin (*actb*) and the Ribosomal protein S18 (*18s*) RNA content in each sample. Gene names follow the accepted nomenclature for zebrafish (http://zfin.org/). The primers used are shown in Table 1. In all cases, each PCR was performed with triplicate samples.

**Table 1:** Primers used for real-time PCR

Gene name	Gene abbreviation	GenBank number	Primer sequences (5´→3´)
Elongation factor 1α	*ef1α*	AF184170	CTGTCAAGGAAATCCGTCGT TGACCTGAGCGTTGAAGTTG
B-actin	*actb*	X89920	GGCACCACACCTTCTACAATG GTGGTGGTGAAGCTGTAGCC
Ribosomal protein S18	*18s*	AM490061	CGAAAGCATTTGCCAAGAAT AGTTGGCACCGTTTATGGTC
Cu/Zn superoxide dismutase	*sod*	AJ937872	CCATGGTAAGAATCATGGCGG CGTGGATCACCATGGTTCTG
Catalase	*cat*	FG264808	TTCCCGTCCTTCATTCACTC CTCCAGAAGTCCCACACCAT
Glutathione reductase	*gr*	AJ937873	CAAAGCGCAGTGTGATTGTGG CCACTCCGGAGTTTTGCATTTC

### Assays of free radical scavenging and antioxidant activity

The free radical scavenging capacity and antioxidant activity of the following samples were determined and compared: muscles of fish fed the experimental diets, muscles of wild gilthead seabream (“wild fish”), a commercial pellet diet, fenugreek seeds powder, seawater from the aquaria of the experimental set-up (“water”) and propyl gallate.

#### Hydrogen peroxide scavenging

Hydrogen peroxide is generated in vivo by several oxidase enzymes and by activated phagocytes. When the samples scavenge the hydrogen peroxide, there is a decrease in the absorption spectrum, and the remaining H_2_O_2_ is measured as the formation of a chromophore recorded at 436 nm using the peroxidase system (Esteban et al. 2014).

#### Hydroxyl radical scavenging

The deoxyribose assay was used to detect possible scavengers of hydroxyl radicals, which are formed by a mixture of ascorbate and FeCl_3_-EDTA. The products of the hydroxyl radical (OH) attack on deoxyribose were measured with thiobarbituric acid (Jiménez et al. 2008a).

#### Measurement of total antioxidant activity by the TEAC assay

This method is based on the inhibition, by antioxidants, of the 2,2-azino-bis-(3-ethylbenzothiazoline-6-sulfonic) (ABTS) radical cation. The Trolox Equivalent Antioxidant Capacity (TEAC) assay measures the ability of antioxidants to quench the ABTS cation in both lipophilic and hydrophilic environments by comparing their scavenging capacity to that of Trolox (Murcia et al. 2009a).

#### Peroxidation of phospholipid liposomes

The ability of samples to inhibit lipid peroxidation was tested using ox brain phospholipid liposomes. The extent of peroxidation was measured as described by Jiménez et al. (2008b).

#### Rancimat test for oxidative stability

Oxidative stability, evaluated by the Rancimat method at 110 ºC (Metrohm model 743, Herisan, Switzerland), reflects the resistance to the development of rancidity in oils/fats. The results are expressed by the protection factor (PF), which is calculated from the induction period (IP, the time in hours until a critical point of oxidation is reached). Samples were macerated with sunflower or olive oil (10% w⁄w) for 3 h at room temperature before analysis (Jiménez et al. 2008a).

#### Determination of antioxidant activity in a linoleic acid system

This method, which is used to determine the antioxidant activity of the samples during storage at unfavourable temperatures (40 °C), measures the inhibition of linoleic acid autoxidation (Esteban et al. 2014).

### Statistical analysis

All measurements were made on three replicates. The results are expressed as mean ± standard error of the mean (SEM). Gene expression data are expressed as fold increase, obtained by dividing each sample value by the mean control value at the same sampling time. Values higher than 1 express an increase, while values lower than 1 express a decrease in the gene concerned. Data were statistically analysed by one-way analysis of variance (ANOVA) to determine differences between groups. The normality of the data was previously assessed using a Shapiro-Wilk test and homogeneity of variance was also verified using the Levene test. Non-normally distributed data were log-transformed prior to analysis and a non-parametric Kruskal-Wallis test, followed by a multiple comparison test when data did not meet parametric assumptions. Statistical analyses were conducted using SPSS 19.0 and differences were considered statistically significant when *p* ≤ 0.05.

## Results

The present work has studied the effects of different diets supplemented with fenugreek seeds on the antioxidant status of farmed gilthead seabream, and also the possible influence of such experimental diets on gilthead seabream growth. After 8 weeks of being fed the diets, all the fish specimens from the group fed the highest dose of fenugreek seeds (10%) showed statistically significant differences (*p* ≤ 0.05), from the control group fish that had received a non-supplemented diet. Supplementation of the diet with fenugreek at 10% significantly increased the growth of fish (Table 2).

**Table 2:** Growth performance of gilthead seabream fed with diets containing 0% (control), 1%, 5% and 10% of fenugreek seeds for 8 weeks

Experimental diets	SGR	WG (%)	CF
Control (0%)	0.631 ± 0.21^a^	42.85 ± 3.11^a^	1.62 ± 0.024^a^
1%	0.666 ± 0.28^ab^	45.22 ± 6.74^ab^	1.63 ± 0.023^a^
5%	0.711 ± 0.18^ab^	48.94 ± 7.89^ab^	1.65 ± 0.022^a^
10%	0.734 ± 0.25^b^	51.90 ± 4.12^b^	1.69 ± 0.026^a^

SGR: Specific growth rate; WG: weight gain; CF: condition factor. Data represent the mean ± SEM (*n* = 20). Different letters denote significant differences between experimental groups (*p* ≤ 0.05)

### Antioxidant status and gene expression

**Fig. 1:**
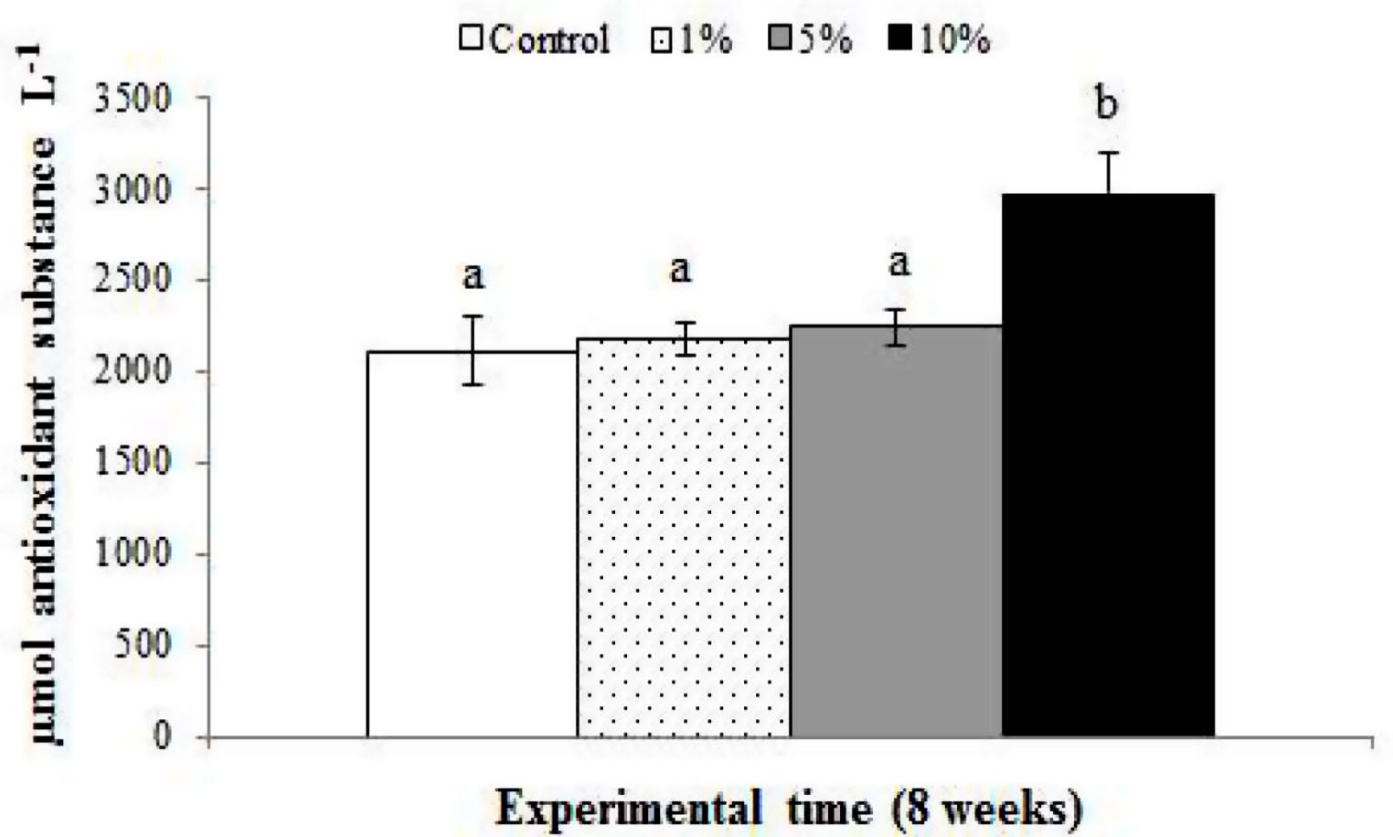
Biological antioxidant potential (expressed as µmol antioxidant substance L^− 1^) of serum of gilthead seabream specimens fed diets containing 0% (control), or 1%, 5% and 10% of fenugreek seeds for 8 weeks. Bars represent the mean ± SEM (*n* = 20). Different letters denote significant differences between experimental groups (*p* ≤ 0.05)

When organisms are exposed to stressors or pollutants, they produce reactive oxygen species (ROS), causing oxidative damage. Animals with high levels of antioxidants, whether constitutive or induced, have been reported to as having greater resistance to such oxidative damage. Antioxidants were analysed in serum from the fish of the control group and fish fed dietary supplementation of fenugreek seeds (Fig. 1). The biological antioxidant potential (BAP) was statistically significantly higher in fish fed the 10% fenugreek-supplemented diets, compared with the values found in the fish fed the control diet.

**Fig. 2:**
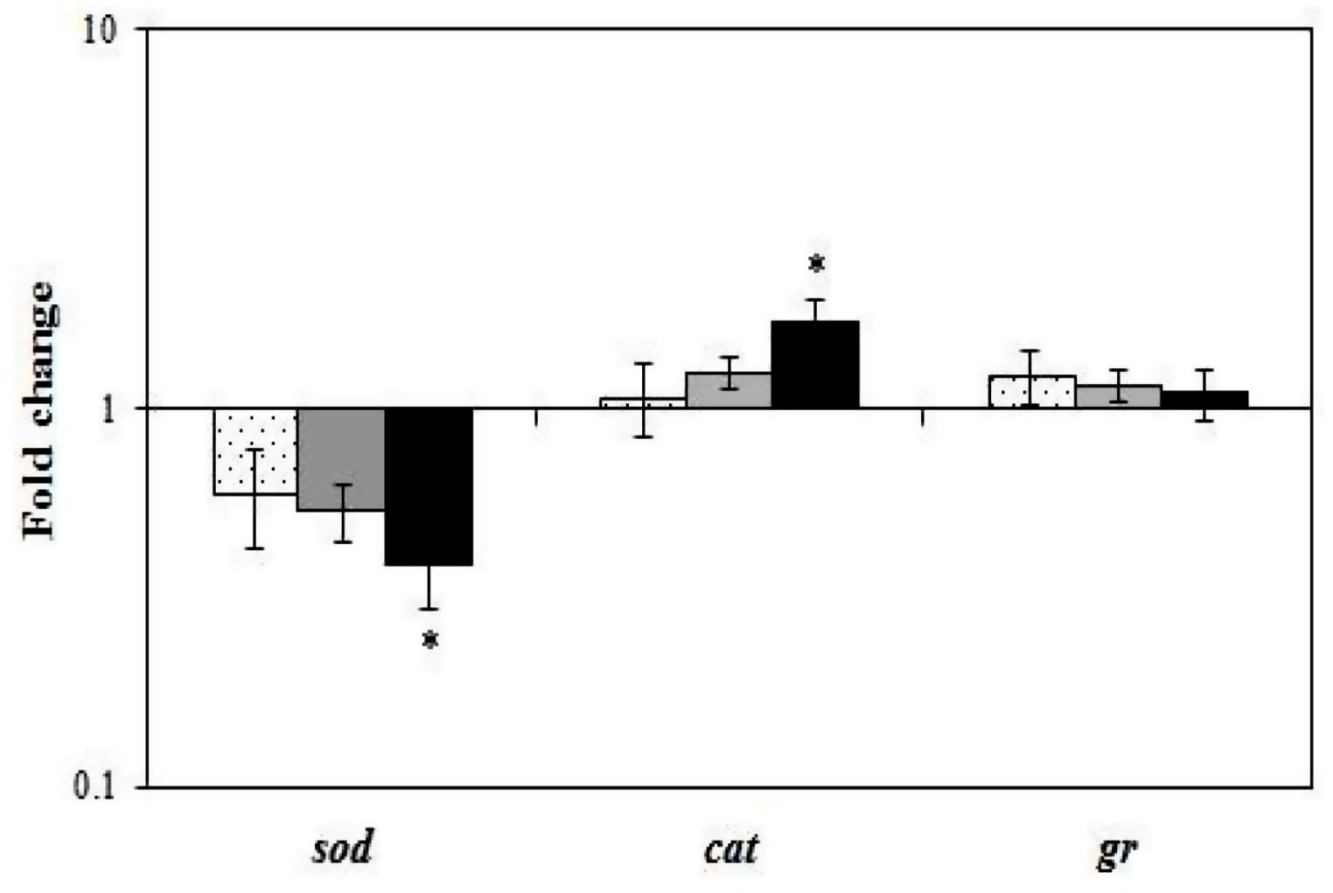
Relative gene expression of antioxidant enzyme genes determined by real-time PCR in liver of gilthead seabream fed with diets containing 0% (control), or 1%, 5% and 10% of fenugreek seeds for 8 weeks. Data are expressed as fold change, obtained by dividing each sample value by the mean control value at the same sampling time. Values higher than 1 express an increase, while values lower than 1 express a decrease in the indicated gene. Bars represent the mean ± SEM (*n* = 20). Asterisks denote significant differences between experimental groups (*p* ≤ 0.05)

Furthermore, to control the level of ROS and to protect cells, fish tissues contain several ROS-scavenging enzymes, among them superoxide dismutase (SOD), catalase (CAT) and glutathione reductase (GR), whose expression levels were monitored in liver from specimens fed the different experimental diets. Dietary supplementation with 10% fenugreek significantly increased the gene expression of the CAT enzyme, since values higher than 1 were detected, indicating greater resistance to oxidative damage. However, SOD enzyme values showed a significant decrease to values lower than 1. Finally, no effect was recorded for the expression of GR; nor were significant differences observed between the fish fed with fenugreek at 1%, 5% and 10% (Fig. 2).

### Free radical scavenging and antioxidant activity

The antioxidant activity of fish fed diets with or without fenugreek supplementation was evaluated by different antioxidant assays and compared with the activity of “wild fish”, the commercial pellet diet, fenugreek seeds, “water” and the common food antioxidant additive, propyl gallate (Table 3). Supplementation of the diet with fenugreek significantly improved (*p* ≤ 0.05) the H_2_O_2_ scavenging capacity, with inhibition percentages of 50–70%. Fish fed the 10% supplemented diet showed similar antioxidant capacity to the “wild fish”, both groups having higher values than the other groups (*p* ≤ 0.05). All the fish samples, analysed (whether or not they were fed fenugreek seeds) and the fenugreek seeds themselves exhibited higher H_2_O_2_ scavenging capacity than propyl gallate (*p* ≤ 0.05).

**Table 3:** Antioxidant activity of samples of gilthead seabream fed with diets containing 0% (control), 1%, 5% and 10% of fenugreek seeds for 8 weeks, wild gilthead seabream, the commercial pellet diet, fenugreek seeds and “water” evaluated by different assays (hydrogen peroxide scavenging, hydroxyl radical scavenging, TEAC, lipid peroxidation and Rancimat test) compared with the activity of a commonly used food antioxidant additive (Propyl gallate)

	Hydrogen peroxide scavenging	Hydroxyl radical scavenging		TEAC†		Lipid peroxidation	Rancimat	
Samples	% Inhibition	% Inhibition	Absorbance (A_532 nm_) Omit ASC ‡	6 min	24 h	% Inhibition	Sunflower oil PF§	Olive oil PF§
**None (control)**		-	0.163 ± 0.002					
Control (0%)	54.6 ± 0.6^d^	50.0 ± 0.7^c^	0.145 ± 0.014	10.1 ± 0.1^bc^	10.7 ± 0.2^ab^	31.1 ± 0.3^e^	0.93 ± 0.02^d^	0.91 ± 0.00^e^
1%	64.9 ± 1.0^bc^	55.6 ± 0.5^b^	0.096 ± 0.001	9.1 ± 0.2^cd^	9.1 ± 0.1^c^	24.8 ± 0.8^ef^	0.97 ± 0.01^d^	0.96 ± 0.01^e^
5%	63.6 ± 0.2^c^	58.0 ± 1.4^b^	0.144 ± 0.043	10.8 ± 0.2^ab^	11.9 ± 0.3^a^	27.7 ± 1.3^ef^	0.99 ± 0.01^cd^	0.96 ± 0.01^e^
10%	72.1 ± 0.4^a^	52.5 ± 1.4^bc^	0.127 ± 0.005	9.9 ± 0.1^bc^	11.3 ± 0.2^ab^	20.0 ± 0.6^ef^	1.09 ± 0.01^b^	1.03 ± 0.01^cd^
wild	68.2 ± 0.8^ab^	69.9 ± 1.0^a^	0.074 ± 0.014	9.7 ± 0.2^c^	10.0 ± 0.5^bc^	47.4 ± 0.9^d^	1.09 ± 0.01^b^	1.09 ± 0.01^bc^
Commercial pellet diet	55.2 ± 1.5^d^	53.8 ± 1.1^bc^	0.165 ± 0.015	8.1 ± 0.2^d^	10.2 ± 0.4^bc^	69.1 ± 0.7^b^	1.09 ± 0.01^b^	1.13 ± 0.01^b^
Fenugreek seeds	67.3 ± 1.4^bc^	57.3 ± 1.5^b^	0.372 ± 0.015	11.5 ± 0.2^a^	11.7 ± 0.1^a^	85.3 ± 0.8^a^	1.09 ± 0.03^b^	1.10 ± 0.00^bc^
“water”	-^f^	-^d^	0.190 ± 0.013	-^e^	-^d^	13.4 ± 1.2^f^	0.96 ± 0.03^d^	0.97 ± 0.00^de^
Propyl gallate	30.9 ± 0.5^e^	-^d^	0.714 ± 0.035	11.5 ± 0.2^a^	11.8 ± 0.1^a^	59.8 ± 0.6^c^	1.30 ± 0.01^a^	1.8 ± 0.03^a^

Wild: wild gilthead seabream

All determinations were performed in triplicate and values show the mean ± SEM. Different uppercase superscript letters indicate significant differences (*p* ≤ 0.05)

† TEAC is the micromolar concentration of a Trolox solution showing the antioxidant capacity equivalent to the dilution of the substance under investigation at 6 min and 24 h

‡ When ascorbic was omitted, the range of absorbance values was lower

§ Values expressed as Protection Factor PF = IP (sunflower or olive oil + sample or additive)/IP (sunflower or olive oil control)

(–) No % inhibition detected

As can be seen, the “wild fish” showed the best (*p* ≤ 0.05) hydroxyl radical scavenging ability (Table 3), while fish fed the diet supplemented with fenugreek (1% and 5%) showed a good antioxidant activity that was significantly higher (*p* ≤ 0.05) with respect to the control fish. However, there were no significant differences in this respect between fish fed 10% fenugreek and fish fed the control diet. The samples lacking ascorbate (Table 3) and exhibiting lower absorbance levels than the control sample were considered as primary antioxidants. However, when the level of the absorbance exceeded that of the control the sample was considered as secondary antioxidant (Murcia et al. 2009b). The fish samples acted as primary antioxidants, while fenugreek seeds and commercial pellet diets were seen to act as secondary antioxidants.

TEAC can be used to provide a ranking order of antioxidants. TEAC is the micromolar concentration of a Trolox solution that has the antioxidant capacity equivalent to the substance under study. In this assay, all fish samples showed TEAC values of around 10 and can be considered as very good ABTS scavengers (6 min) (Table 3). The TEAC after 24 h showed similar results. Fenugreek showed the best TEAC value, and did not show significant differences from propyl gallate (*p* ≤ 0.05), similarly to the findings of Kaviarasan et al. (2007), who observed a substantial fall in the absorbance of ABTS radical in the presence of fenugreek seeds.

One way to test the antioxidant ability of foods is to examine whether it inhibits the peroxidation of lipid systems by scavenging peroxyl radicals. The formation of peroxyl radicals is the most important step in lipid peroxidation, although the radicals can also be formed in non-lipid systems (Murcia et al. 2009a). The results pointed to three different antioxidant levels: the very good lipoperoxyl radical scavenging capacity of fenugreek seeds, which showed significant differences (*p* ≤ 0.05) from the rest of the samples (Table 3); good lipoperoxyl radical scavenging capacity for the commercial pellet diet and “wild fish” (*p* ≤ 0.05), and an intermediate level for the fish fed (or not) with fenugreek. All the samples were also compared with propyl gallate, and only fenugreek and the commercial pellet diet exhibited higher antioxidant activity, the differences being statistically significant (*p* ≤ 0.05).

The relative activity of an antioxidant is expressed by the PF, which is calculated by dividing the IP of oil containing added antioxidants by the IP of the control oil with no additives (Table 3). Fish fed 10% fenugreek, “wild fish”, the commercial pellet diet, fenugreek and propyl gallate showed PF values above 1, meaning that they protect the oil from oxidation. However, the PF results were lower for the rest of the samples, with significant differences (*p* ≤ 0.05).

**Fig. 3:**
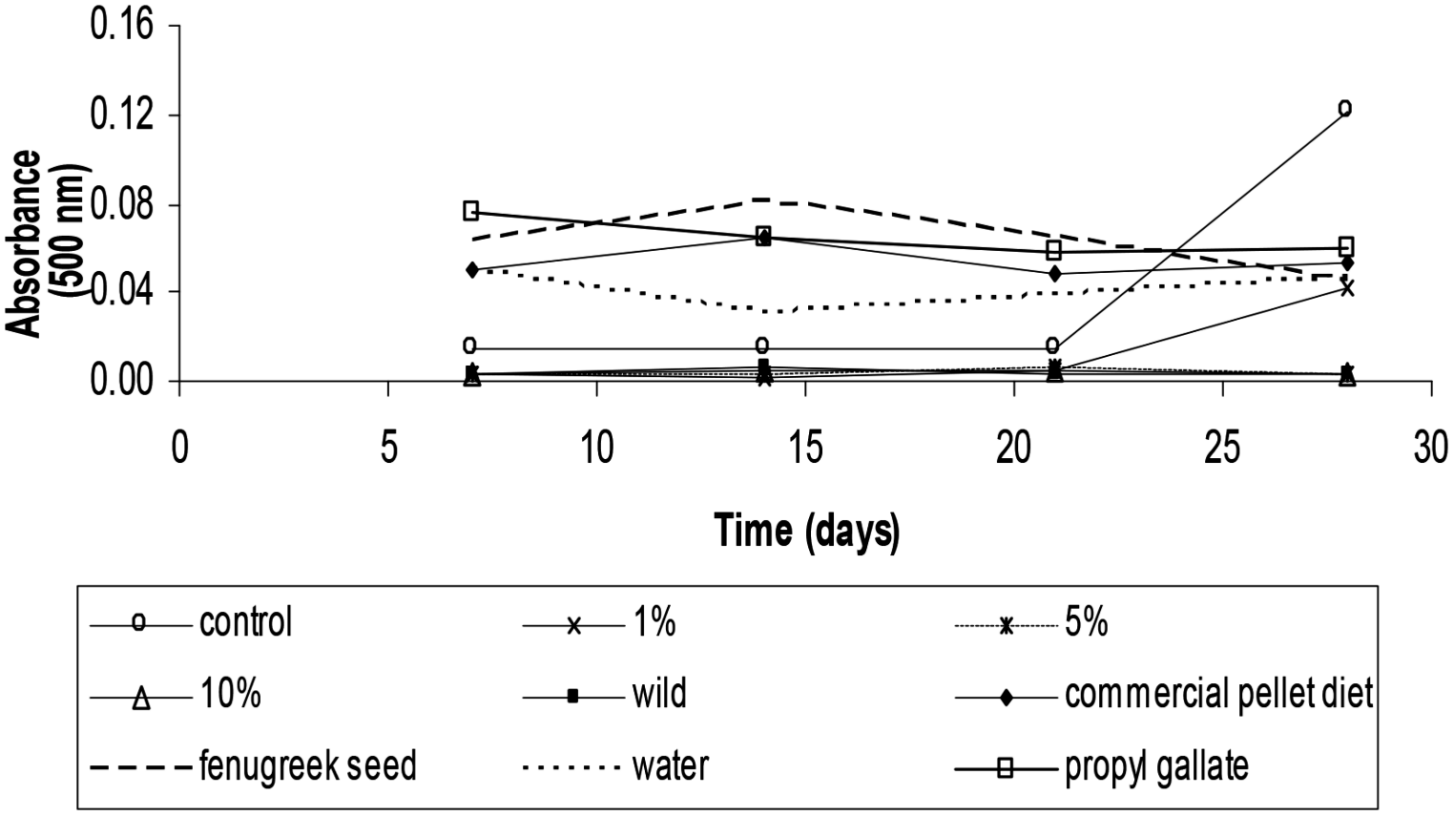
Evolution of the absorbance at 500 nm for the oxidation of linoleic acid in the presence of samples of gilthead seabream fed diets containing 0% (control), or 1%, 5% and 10% of fenugreek seeds for 8 weeks, “wild fish”, a commercial pellet diet, fenugreek seeds and “water” compared with activity of common feed additive antioxidant (propyl gallate) during 28 days of storage

Finally, the antioxidant activity was determined by the linoleic acid system. The absorbance values obtained for the autoxidation of linoleic acid during 28 days of storage are shown in Fig. 3. According to the statistical analyses, these results can be divided into two groups, with significant (*p* ≤ 0.05) differences between them. The first group included all the fish analysed with high antioxidant activity (more than 95% inhibition during storage), while the second group included propyl gallate, the pellet diet, fenugreek and “water”, which exhibited a good level of antioxidant activity (around 80%) after 28 days of storage.

## Discussion

Herbal plants and their products are widely used in many countries of the world as they are considered as model feed additives due to the absence of residual effects and their ability to positively influence numerous physiological processes. The present study looks at the possible use of fenugreek seeds as an additive for fish fed. Botanically, fenugreek (*T. foenum-graecum* L.) is an annual leguminous herb of the family Fabaceae. This species is cultivated in Europe, India, Turkey, China, Canada and Northern Africa (U.S. National Plant Germplasm System). Fenugreek is used as a herb (both dried and fresh leaves), spice (seeds), and as a vegetable (fresh leaves, sprouts and microgreens). As regards their medical uses, fenugreek seeds (which have a strong fragrance) have long been used in traditional medicine as a laxative, digestive, and as a remedy for cough and bronchitis. They may also help control cholesterol, triglycerides and high blood sugar levels in diabetics (Sfar et al. 2018). The antioxidant properties of germinated fenugreek seeds have been attributed to the presence of flavonoids and polyphenols (Dixit et al. 2005).

Fenugreek seeds have a high protein content (around 20–30%), and are rich in important free amino acids such as lysine and tryptophan. Fenugreek seeds also contain 45–60% carbohydrates, a small amount of oils (5–10%) and many other components such as alkaloids (mostly trigonelline), flavonoids, sapogenins, vitamins and volatile oils. Furthermore, fenugreek seeds are a source of minerals, including copper, potassium, calcium, iron, selenium, zinc, manganese and magnesium (Awad and Austin 2010; Hudec et al. 2007; Mayer and Lehmann 2000).

The results of the present study showed that the group of fish fed the highest amount of fenugreek grew more than the other experimental groups. Taking into account the composition of fenugreek seeds, it may be assumed that the growth improvement observed fish fed the supplemented diets was due to the seeds. However, further research is needed in order to improve the digestibility of the diet by fish.

Some vitamins are indispensable antioxidant nutrients necessary for optimal growth, development, and reproduction in animals (Traber and Stevens 2011). In fact, several synthetic antioxidants are authorized for use as fish feed additives in the EU (Lundebye et al. 2010). However, there is growing interest in the search for new sources of natural antioxidants, as an alternative strategy to prevent oxidative damage in various health disorders, in which oxidative stress is known to play a part. According to the bibliography consulted, studies focusing on the immunostimulant properties of natural products in fish (reviewed by Elsayed et al. 2015) are more numerous than those focusing on their antioxidant properties. The results presented here confirm that the gene expression of the main enzymes related with oxidative stress was affected in the liver of the gilthead seabream following the inclusion of fenugreek seeds in the diet.

Superoxide dismutase, a major scavenger of O2•-, catalyses the conversion of superoxide radical into hydrogen peroxide (H_2_O_2_). The H_2_O_2_ is then scavenged by CAT and different classes of peroxidases, while GR plays a key role in the ascorbate-glutathione cycle and seems to remove the excess of H_2_O_2_. A relationship between the catalase level and H_2_O_2_ scavenging was established, levels being higher in fish fed the highest fenugreek supplemented diet than in fish fed control diet. It has also been suggested that catalase could function as a potent antioxidant enzyme and might even play a role in post-immune responses with respect to the peroxidase activity (Elvitigala et al. 2013).

The antioxidant activity of the bioactive compounds of fenugreek could be due to their ability to scavenge free radicals, donate hydrogen atoms or electrons, or chelate metal ions; in this way, these compounds are incorporated into cell membranes, protecting the tissues from oxidation by reactive oxygen species (Descalzo and Sancho 2008; Singh et al. 2014). Hydroxyl radicals, which are highly reactive, can be generated in the human body under physiological conditions, although the compound can also be generated from peroxyl radicals (Murcia et al. 2009b). Fish fed 1%, 5%, and 10% fenugreek showed good hydroxyl radical scavenging ability, which suggests that the fenugreek depressed hydroxyl radical activity and improved fish resistance to oxidative stress. When ascorbate was omitted, the attack on deoxyribose was less intense, because the absence of ascorbate decreases the concentration of OH**·** in the reaction mixture, differentiating primary from secondary antioxidants (Murcia et al. 2009b).

Similar to our observations, Mohseni and Ozorio (2014) found antioxidant activity to be higher in fish fed a lower concentration of supplement in the diet. The higher hepatic TBARS values observed in fish fed low and high concentrations of dietary L-carnitine than in those fed a suitable amount. L-carnitine indicated that both insufficient and excess dietary L-carnitine induced oxidative stress in beluga. In the same study, the hepatic TBARS values showed an inverse trend from that of hepatic SOD activity. The high hepatic TBARS values in fish fed both insufficient and excess dietary L-carnitine might be due to low SOD activity. It is suggested then, that excess L-carnitine stored in the liver causes high oxidative stress and destroys enzyme activity.

The wild fish showed good lipoperoxyl radical scavenging capacity, which agrees with those of Valente et al. (2011), who found that wild fish had much lower lipid content than their farmed counterparts. The medium-level activity observed in fish could be due to the presence of high amounts of PUFA, iron and heme, which would make fish muscle highly susceptible to lipid oxidation (Maqsood et al. 2014). Simat et al. (2012) observed the same difference in rancidity between wild and farmed fish, due to the lipid content. It is interesting to note that in fish fed 10% fenugreek seeds the PF values increased, oxidative stability being similar to that observed in wild fish.

The good level of antioxidant activity found in all fish using the linoleic acid system could be due to the proximate composition, since amino acids in fish are known to possess significant antioxidant properties, generally functioning as synergists or primary antioxidants (Kikugawa et al. 1990). Yamashoji et al. (1979) found that most amino acids had a significant antioxidant potential in the linoleic acid model system.

## Conclusion

After feeding gilthead seabream specimens with fenugreek-supplemented diets for eight weeks fish growth and antioxidant status (ROS scavenging, catalase and oil oxidative stability) were seen to have improved compared with the control, suggesting that increasing the levels of antioxidants could be a solution to reducing oxidative stress, as mentioned by Poljsak (2011). The present results strongly show that fenugreek could be considered as a functional food ingredient for farmed fish.

## Data Availability

Not applicable.
